# Supplementary material for: Resolving the full spectrum of human genome variation using Linked-Reads
Source: Genome Res. 2019 Apr;29(4):635–45. doi: 10.1101/gr.234443.118 (PMC6442396; doi:10.1101/gr.234443.118)
Supplement: Supplemental Material [file supp_gr.234443.118_Supplemental_longranger-2.2.zip › longranger-2.2.tar.bz2!/lr2.2-public/lib/stan/cmdstan/stan/doc/api/html/whitespace__grammar_8hpp_source.html]

Stan: src/stan/gm/grammars/whitespace\_grammar.hpp Source File


|  |  |
| --- | --- |
|  | Stan  2.5.0  probability, sampling & optimization |


- Main Page
- Related Pages
- Namespaces
- Classes
- Files

- File List
- File Members

All Classes Namespaces Files Functions Variables Typedefs Enumerations Enumerator Friends Macros Pages

- src
- stan
- gm
- grammars

whitespace\_grammar.hpp

Go to the documentation of this file.

1 #ifndef STAN\_\_GM\_\_PARSER\_\_WHITESPACE\_GRAMMAR\_HPP

2 #define STAN\_\_GM\_\_PARSER\_\_WHITESPACE\_GRAMMAR\_HPP

3

4 #include <boost/spirit/include/qi.hpp>

5

6 namespace stan {

7

8  namespace gm {

9

10  template <typename Iterator>

11  struct whitespace\_grammar

12  : public boost::spirit::qi::grammar<Iterator> {

13  public:

14  whitespace\_grammar();

15  // private:

16  boost::spirit::qi::rule<Iterator> whitespace;

17  };

18

19

20  }

21

22 }

23

24

25

26 #endif

stan::gm::whitespace\_grammar::whitespace

boost::spirit::qi::rule< Iterator > whitespace

**Definition:** whitespace\_grammar.hpp:16

stan::gm::whitespace\_grammar

**Definition:** whitespace\_grammar.hpp:11

stan::gm::whitespace\_grammar::whitespace\_grammar

whitespace\_grammar()

**Definition:** whitespace\_grammar\_def.hpp:13


---

[ Stan Home Page ]

*© 2011–2014,
Stan Development Team.*
